# Rapid Influx and Death of Plasmacytoid Dendritic Cells in Lymph Nodes Mediate Depletion in Acute Simian Immunodeficiency Virus Infection

**DOI:** 10.1371/journal.ppat.1000413

**Published:** 2009-05-08

**Authors:** Kevin N. Brown, Viskam Wijewardana, Xiangdong Liu, Simon M. Barratt-Boyes

**Affiliations:** 1 Department of Infectious Diseases and Microbiology, Graduate School of Public Health, University of Pittsburgh, Pittsburgh, Pennsylvania, United States of America; 2 Center for Vaccine Research, School of Medicine, University of Pittsburgh, Pittsburgh, Pennsylvania, United States of America; NIH/NIAID, United States of America

## Abstract

Plasmacytoid dendritic cells (pDC) are essential innate immune system cells that are lost from the circulation in human immunodeficiency virus (HIV)–infected individuals associated with CD4^+^ T cell decline and disease progression. pDC depletion is thought to be caused by migration to tissues or cell death, although few studies have addressed this directly. We used precise methods of enumeration and *in vivo* labeling with 5-bromo-2′-deoxyuridine to track recently divided pDC in blood and tissue compartments of monkeys with acute pathogenic simian immunodeficiency virus (SIV) infection. We show that pDC are lost from blood and peripheral lymph nodes within 14 days of infection, despite a normal frequency of pDC in bone marrow. Paradoxically, pDC loss masked a highly dynamic response characterized by rapid pDC mobilization into blood and a 10- to 20-fold increase in recruitment to lymph nodes relative to uninfected animals. Within lymph nodes, pDC had increased levels of apoptosis and necrosis, were uniformly activated, and were infected at frequencies similar to CD4^+^ T cells. Nevertheless, remaining pDC had essentially normal functional responses to stimulation through Toll-like receptor 7, with half of lymph node pDC producing both TNF-α and IFN-α. These findings reveal that cell migration and death both contribute to pDC depletion in acute SIV infection. We propose that the rapid recruitment of pDC to inflamed lymph nodes in lentivirus infection has a pathologic consequence, bringing cells into close contact with virus, virus-infected cells, and pro-apoptotic factors leading to pDC death.

## Author Summary

Plasmacytoid dendritic cells (pDC) are essential components of the innate immune system whose loss from blood is associated with disease progression in human immunodeficiency virus–infected individuals. The mechanism of pDC loss is undefined but is believed to be associated with migration to tissues or cell death. To address this question, we studied pDC kinetics in blood and tissues in the related rhesus macaque monkey model of simian immunodeficiency virus infection. We found that pDC were present in normal numbers in bone marrow but were lost from blood and lymph nodes within 14 days of intravenous infection. Underlying pDC loss was a profound mobilization of pDC from bone marrow into blood and subsequent influx into lymph nodes. In lymph nodes pDC were activated, apoptotic, and frequently infected with virus. Nevertheless, pDC were functionally normal with respect to cytokine production. We conclude that migration and death both contribute to pDC depletion, with influx into lymph nodes bringing cells into an environment favoring their death by infection or apoptosis.

## Introduction

Plasmacytoid dendritic cells (pDC) are important in bridging innate and adaptive immune responses to pathogens [1]–[3]. pDC derive from bone marrow and are recruited into lymph nodes in response to inflammation, producing type I interferon (IFN) and contributing to the generation of virus-specific T cell responses [2]–[5]. Human immunodeficiency virus (HIV) infection of humans and simian immunodeficiency virus (SIV) infection of monkeys result in pDC loss from blood that is correlated with high viral load and reduced numbers of CD4^+^ T cells [6]–[16]. pDC-derived type I IFN limits HIV replication in CD4^+^ T cells [17]–[19] and frequencies of pDC are higher in HIV-infected long-term non-progressors than healthy donors [20], suggesting that diminished pDC numbers likely contribute to the lack of immune control in this disease. However, the mechanism of pDC loss in HIV infection has not been defined.

A central hypothesis accounting for pDC depletion from blood during HIV infection is recruitment to inflamed lymphoid tissues [18],[21]. Evidence for pDC recruitment comes from the finding that pDC exposed to HIV express the chemokine receptor CCR7 required for homing to lymph node paracortex [21], and recent data in rhesus macaques confirm that CCR7 is induced on circulating pDC in response to pathogenic SIV infection [22]. Moreover, expression of CXCL9 is highly upregulated in lymphoid tissues in acute SIV infection [23],[24], and pDC enter lymph nodes in a CXCL9-dependent manner [4]. However, there is also substantial evidence to support a role for cell death in pDC loss in HIV infection. HIV infects pDC both *in vitro* and *in vivo* and induces cytopathic effects and death following maturation [25]–[29]. In addition, pDC from HIV-infected individuals undergo death by apoptosis and necrosis following interaction and fusion with HIV-infected cells [17]. The relative contribution of recruitment to tissues and cell death to pDC loss in HIV infection remains to be determined.

To tease out the different potential mechanisms of pDC loss, we studied kinetics of the pDC response during acute infection of rhesus macaques with the pathogenic SIV strain SIVmac251. We enumerated pDC in different compartments and used *in vivo* labeling with the thymidine analogue 5-bromo-2′-deoxyuridine (BrdU) together with Ki-67 staining to document mobilization and recruitment of recently divided cells that would otherwise be obscured by net cell loss. We find that pDC are lost from blood and peripheral lymph nodes early in SIV infection despite massive mobilization and recruitment, associated with pDC activation, infection and death in lymph nodes. These findings demonstrate that pDC recruitment and killing work together in the context of the lymph node to bring about pDC depletion in SIV infection.

## Results

### Rapid loss of pDC from blood and lymph node during acute SIV infection

**Figure 1:**
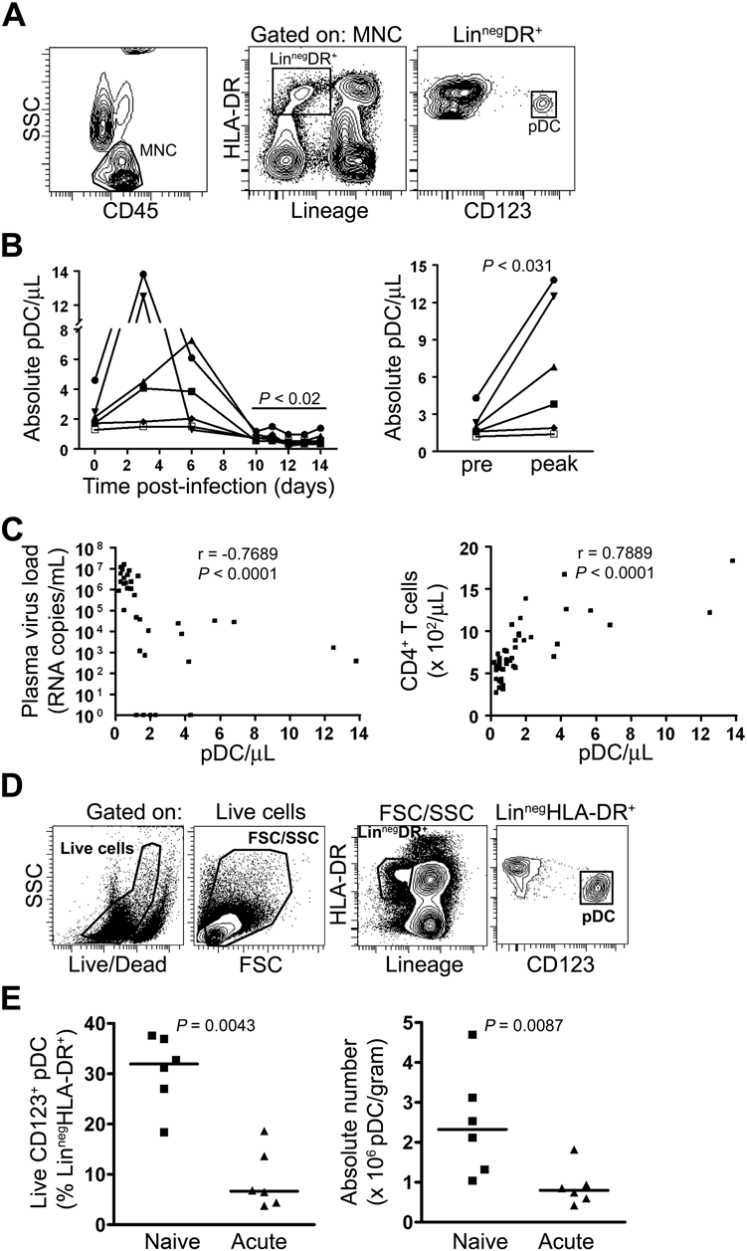
pDC are transiently increased and then lost from blood and lymph nodes in acute SIV infection. (A) Representative contour plots demonstrating the gating strategy used to define pDC within the mononuclear cell fraction (MNC) of freshly isolated peripheral blood leukocytes. (B) Absolute number of pDC in blood during acute SIV infection over all days (left) and comparing pre-infection to peak responses at day 3 or 6 (right). Symbols represent individual animals with the baseline number of pDC/µL blood prior to infection (day 0) representing the median of 4 independent measurements for each animal. (C) The number of pDC in blood was inversely correlated with viral load and positively correlated with CD4^+^ T cell counts during the 14-day period. (D) Representative plots demonstrating the gating strategy used to delineate the Lineage^−^ HLA-DR^+^ cells and CD123^+^ pDC in axillary lymph node cell suspensions. (E) Number of live pDC in lymph nodes of naïve animals and animals at 14 days post-infection expressed as percent of pDC within the Lineage^−^ HLA-DR^+^ fraction (left) and as absolute number of pDC/g of tissue (right). Symbols represent individual animals and horizontal lines represent medians.

In order to document changes in pDC number in blood during HIV infection, we used the rhesus macaque model of intravenous infection with the pathogenic isolate SIVmac251. Infection resulted in detectable viremia within 3 days of infection that plateaued at day 12 and was associated with an expected decline in the number of blood CD4^+^ T cells by day 10 (Figure S1). pDC were identified within the CD45^+^ mononuclear cell fraction of peripheral blood leukocytes (PBL) as Lineage (CD3, CD14, CD20)^−^ HLA-DR^+^ CD123^+^ cells using flow cytometry (Figure 1A) [13]. pDC determinations were made by multiplying the percentage of pDC in this CD45^+^ mononuclear cell fraction by the absolute number of CD45^+^ mononuclear cells in whole blood determined using TruCOUNT tubes [30]. In contrast to CD4^+^ T cells, there was a significant increase in the number of blood pDC between 3 and 6 days post infection, in some instances reaching 7 times that of pre-infection levels, indicative of peracute mobilization of pDC (Figure 1B). This was followed at day 10 by a significant decrease in pDC number from baseline which remained suppressed until at least day 14 (Figure 1B), when CD4^+^ T cells had begun to recover (Figure S1). The absolute number of pDC in blood was inversely correlated with plasma viral load and positively correlated with CD4^+^ T cell number during the course of primary SIV infection (Figure 1C). To determine whether the drop in pDC from blood at days 10–14 of infection was associated with an increase in pDC number in lymph nodes, we harvested axillary lymph nodes prior to and 14 days after infection and determined the proportion and absolute number of pDC in cell suspensions. pDC were identified as CD123^+^ cells within the Lineage^−^ HLA-DR^+^ fraction following exclusion of dead cells based upon staining with an amine-reactive viability dye. This gate excludes the majority of myeloid DC (mDC) which express high levels of HLA-DR and have significant autofluorescence that places them above the Lineage^+^ HLA-DR^+^ population (Figure 1D) [13]. Surprisingly, the percentage of live pDC within the Lineage^−^ HLA-DR^+^ gate decreased significantly from a median of 32% prior to infection to 7% at day 14 post infection (Figure 1E). Similarly, the absolute number of live pDC decreased by more than 65% from a pre-infection median of 2.3×10^6^ cells/g to a post infection median of 0.8×10^6^ cells/g (Figure 1E). This decrease was not offset by lymph node hyperplasia as the total cellularity of lymph nodes actually decreased significantly in response to infection, from a median of 5.5×10^8^ cells/g (range 3.7×10^8^ to 8.3×10^8^) to 3.3×10^8^ cells/g (range 2.4×10^8^ to 4.3×10^8^; *P* = 0.04 by Mann-Whitney U test). This loss in cellularity is likely the combined effect of tissue edema and CD4^+^ T cell depletion [31]. These findings indicate that acute SIV infection results in a bimodal pDC response in blood, with rapid pDC increases followed by loss, and that this loss is paralleled by depletion of pDC from lymph nodes.

### Acute SIV infection does not alter the frequency of pDC in bone marrow

**Figure 2:**
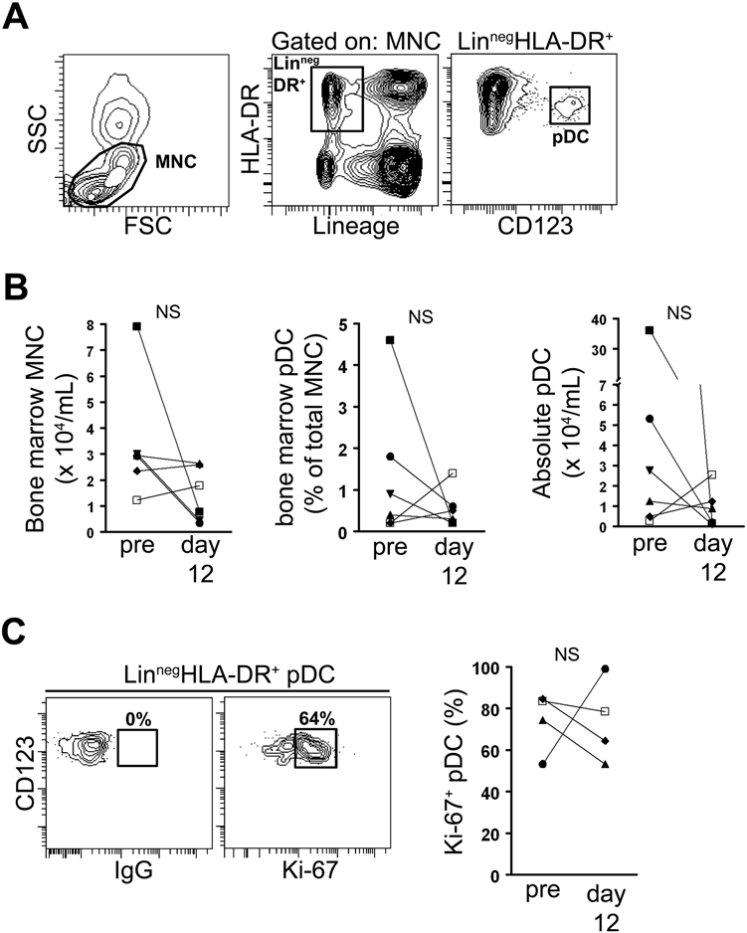
Normal frequency of pDC in bone marrow in acute SIV infection. (A) Representative contour plots demonstrating the gating strategy used to define pDC within the mononuclear cell (MNC) fraction of bone marrow aspirates. (B) Paired analyses of the absolute number of MNC in bone marrow (left), the frequency of pDC within the total MNC fraction (middle) and the absolute number of pDC in bone marrow (right) before and 12 days after SIV infection. Symbols represent individual animals. (C) Left: pDC were gated as in (A) and the percentage of pDC expressing Ki-67 determined by comparing to staining with an isotype control Ab. Contour plots represent an analysis at day 12 post infection and numbers represent the percentage of cells within the indicated gates. Right: Paired analysis of the percentage of Ki-67^+^ pDC before infection and at day 12 post-infection. NS = not significant.

The depletion of pDC from both blood and lymph nodes during acute SIV infection could be explained by bone marrow suppression leading to a loss of pDC production. To address this possibility we analyzed the frequency of phenotypically-defined pDC in paired bone marrow samples taken prior to and 12 days following SIV infection. pDC were identified within the Lineage^−^ HLA-DR^+^ fraction of bone marrow mononuclear cells by expression of CD123 (Figure 2A), as has previously been described [32]. There was inherent variability in the samples collected from bone marrow as the total number of mononuclear cells and the proportion and number of pDC varied widely between animals. However, there was no significant difference in these parameters prior to and after infection when animals were compared as a group (Figure 2B). We next analyzed bone marrow pDC for expression of the nuclear proliferation antigen Ki-67, which is expressed by cells in a non-G_0_ phase of the cell cycle and thus provides an approximation of the recent proliferative history of a given cell population [33]. The majority of bone marrow pDC in SIV-naïve animals were Ki-67^+^ reflecting a high rate of production [5] and this was unchanged as a result of SIV infection (Figure 2C). These data indicate that acute SIV infection does not significantly alter pDC production or dynamics in bone marrow.

### Rapid mobilization of pDC into blood during acute SIV infection

**Figure 3:**
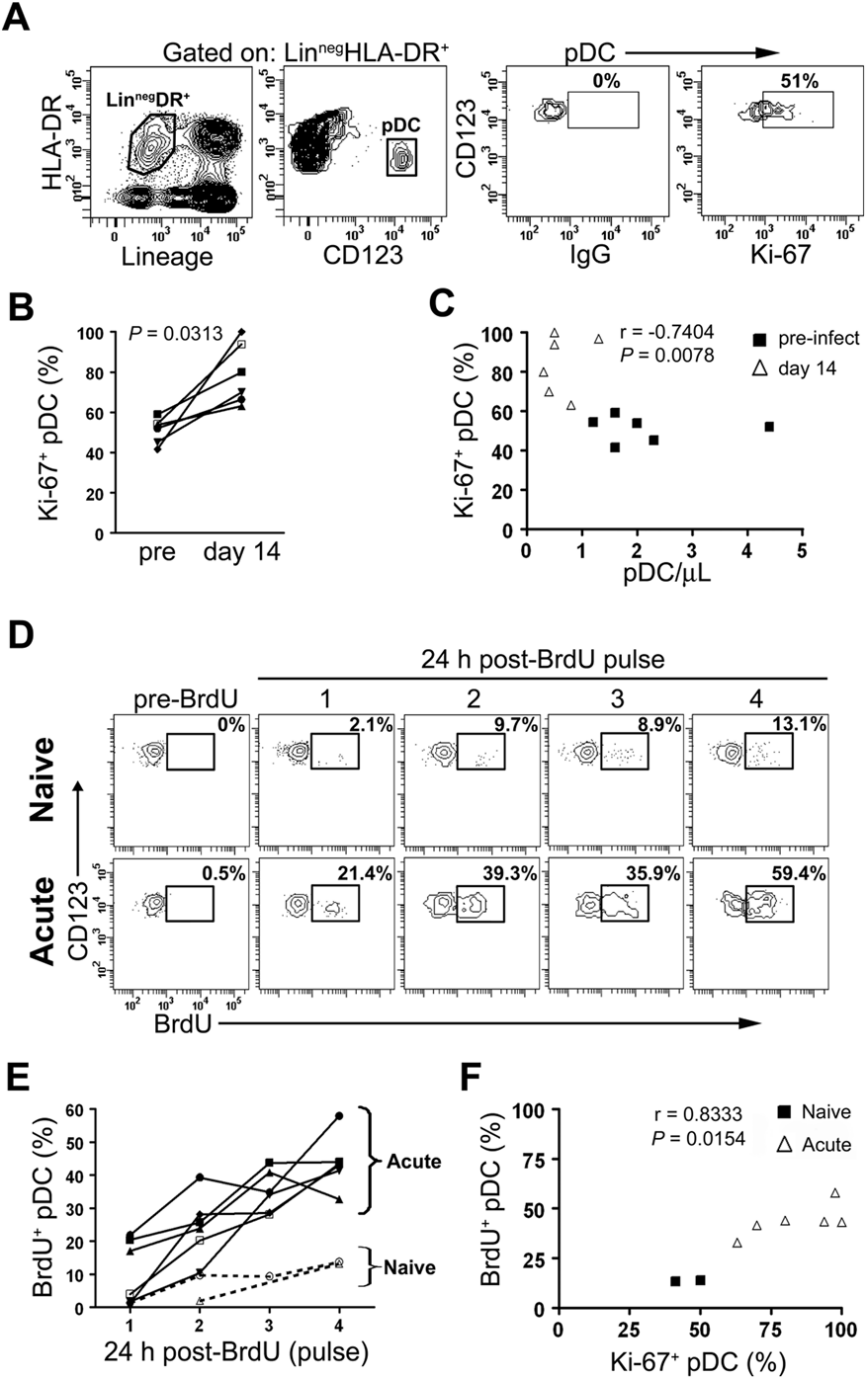
Rapid mobilization of pDC into blood during acute SIV infection. (A) Representative contour plots from an animal prior to infection demonstrating the gating strategy used to identify Ki-67^+^ pDC in blood. pDC were gated as in Figure 1 and Ki-67 positivity determined based on the level of background fluorescence using the isotype-matched control Ab. Numbers represent the percentage of cells within the indicated gates. (B) Paired analysis of percentage of Ki-67^+^ pDC in blood prior to infection and at day 14 after infection. Symbols represent individual animals. (C) The absolute number of pDC in blood was inversely correlated with the percentage of cells expressing Ki-67. Shown are data from individual animals prior to infection and 14 days after infection. (D) Representative contour plots demonstrating the gating strategy used to identify BrdU^+^ pDC prior to and 24-hours after four consecutive intravenous daily injections of BrdU in an SIV-naive and SIV-infected macaque. BrdU was delivered starting on day 10 of infection. Numbers represent the percentage of BrdU^+^ pDC within each gate. (E) The percent of BrdU^+^ pDC in blood of SIV-naïve animals (dashed lines) and SIV-infected animals (solid lines) 24 hours after each BrdU pulse. (F) The percent of BrdU^+^ pDC was positively correlated with the percent of Ki-67^+^ pDC 24 hours after the final BrdU administration.

The rapid but transient increase in the number of pDC in blood together with normal pDC frequencies in bone marrow raised the possibility that pDC were being actively mobilized from bone marrow into blood during acute SIV infection. To measure pDC mobilization directly we again turned to markers of cell division, based on findings in the mouse that pDC do not undergo proliferation in peripheral tissues under physiologic conditions [5]. We first needed to confirm that pDC in the rhesus macaque had the same characteristics, especially given that exposure to SIV can induce marked pDC activation [16],[22] that conceivably could result in cell division. To address this we stimulated normal macaque peripheral blood mononuclear cells (PBMC) with 3M-007, an agonist of Toll-like receptor (TLR)7/8, as viral RNA stimulates pDC largely through TLR7 engagement [22],[34]. Cells were then pulsed with BrdU and pDC analyzed by flow cytometry for BrdU incorporation. No BrdU incorporation was detected indicating that pDC were not induced to enter S-phase of the cell cycle following *in vitro* activation (Figure S2). pDC are likely to be exposed to a wide range of microbial and inflammatory stimuli in addition to viral RNA as a consequence of SIV infection [35]. To determine whether such *in vivo* activation results in pDC proliferation we incubated lymph node cells harvested from a rhesus macaque 14 days after SIVmac251 infection with BrdU and examined cells for BrdU incorporation. There was no detectable BrdU incorporation by pDC, indicating lack of cell division (Figure S2). In contrast, 15% of lymph node mDC incorporated BrdU (Figure S2), consistent with findings in the mouse that mDC undergo proliferation in lymphoid tissues [5]. These findings confirm that rhesus macaque pDC do not undergo detectable proliferation in the blood or lymph node compartments either in health or during acute SIV infection. Using this information, we then analyzed blood pDC from our cohort of animals for expression of Ki-67, which would provide an indication of the pDC fraction that was recently mobilized. Prior to SIV infection, the mean percentage of Ki-67^+^ pDC was 52% (Figure 3A and 3B), indicating that half of all pDC in the circulation were recently released from bone marrow. However, by day 14 post infection 87% of circulating pDC expressed Ki-67 indicative of mobilization (Figure 3B), and this increase was inversely correlated with the absolute number of blood pDC (Figure 3C). To accurately determine the rate of mobilization of pDC into blood we administered BrdU by intravenous injection at days 10, 11, 12 and 13 post infection and sampled blood 24 hours after each administration. Any BrdU^+^ pDC in blood would be marked as having been released from bone marrow since the time of the BrdU pulse. In SIV-naïve monkeys only 2% of blood pDC were BrdU^+^ at 24 hours after the first pulse, reaching a maximum of 13% after the fourth pulse, indicating a relatively low rate of pDC mobilization in health (Figure 3D and 3E). In stark contrast, upwards of 20% of blood pDC from SIV-infected animals at day 11 were BrdU^+^ after a single BrdU pulse and this approached 60% after the fourth pulse at day 14 post infection (Figure 3D and 3E). The degree of Ki-67 staining and BrdU incorporation at day 14 were strongly correlated (Figure 3F). These findings indicate that by day 10 post infection rhesus macaques had a profound mobilization of pDC from bone marrow that was effectively masked by declining cell numbers in blood.

### Massive recruitment of pDC into peripheral lymph nodes during acute SIV infection

**Figure 4:**
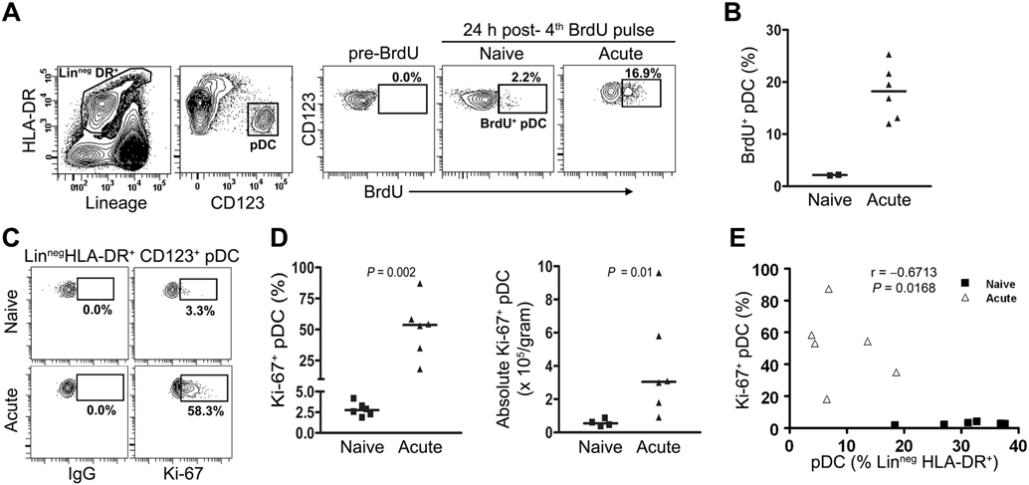
Massive recruitment of recently mobilized pDC to peripheral lymph nodes in acute SIV infection. (A) Representative plots demonstrating the gating strategy used to identify pDC in CD20-depleted axillary lymph nodes (left) and BrdU staining of pDC in a SIV-naïve and SIV-infected macaque (right). Numbers represent the percentage of cells within the indicated gate. (B) Percent of BrdU^+^ pDC in lymph nodes 24 hours after the final BrdU pulse in naïve and infected animals. Symbols represent individual animals and horizontal bars represent medians. (C) Representative plots demonstrating Ki-67 staining in lymph node pDC as gated in (A). (D) Number of Ki-67^+^ pDC in lymph nodes from SIV-naive animals and animals at day 14 post-infection expressed as a percent of all pDC (left) and as absolute number/gram of tissue (right). Symbols represent individual animals and horizontal bars represent medians. (E) The frequency of pDC within the Lin^−^ HLA-DR^+^ gate is inversely correlated with the percent of Ki-67^+^ pDC in lymph nodes. Symbols represent individual SIV-naive animals and animals at 14 days post infection.

We next used BrdU incorporation and Ki-67 staining to investigate whether increased mobilization of pDC into blood was paralleled by recruitment of pDC to lymph nodes, despite the loss of pDC from these tissues. Axillary lymph nodes were harvested from SIV-infected monkeys at day 14 post infection and from SIV-naïve monkeys receiving the same course of BrdU and pDC were analyzed for BrdU incorporation (Figure 4A). Only 2% of lymph node pDC were BrdU^+^ after 4 pulses of BrdU in SIV-naïve monkeys, indicating a low level of pDC recruitment into normal lymph nodes. However, 19% of lymph node pDC from SIV-infected monkeys were BrdU^+^, representing a ∼10-fold increase in pDC recruitment over the 4-day labeling period relative to controls (Figure 4B). Strikingly, the mean proportion of lymph node pDC that were Ki-67^+^ in SIV-naïve monkeys was only 2.5%, whereas more than 50% of lymph node pDC from monkeys at day 14 post infection stained for Ki-67. This represents a 20-fold increase in recently-divided pDC within lymph nodes as a result of SIV infection (Figure 4C and 4D). In fact, whereas the total number of lymph node pDC declined in acute SIV infection (Figure 1E) the number of Ki-67^+^ pDC in lymph nodes was significantly increased over SIV-naïve lymph nodes (Figure 4D). Nevertheless, the proportion of pDC that expressed Ki-67 had a strong inverse correlation with the total number of pDC in lymph nodes, indicating that recruitment and loss were occurring concurrently (Figure 4E). Together, these data demonstrate that pDC undergo a marked recruitment to peripheral lymph nodes during acute SIV infection but that the net result is an overall loss of pDC from these tissues.

### Activation, infection, and death of pDC in lymph nodes during acute SIV infection

**Figure 5:**
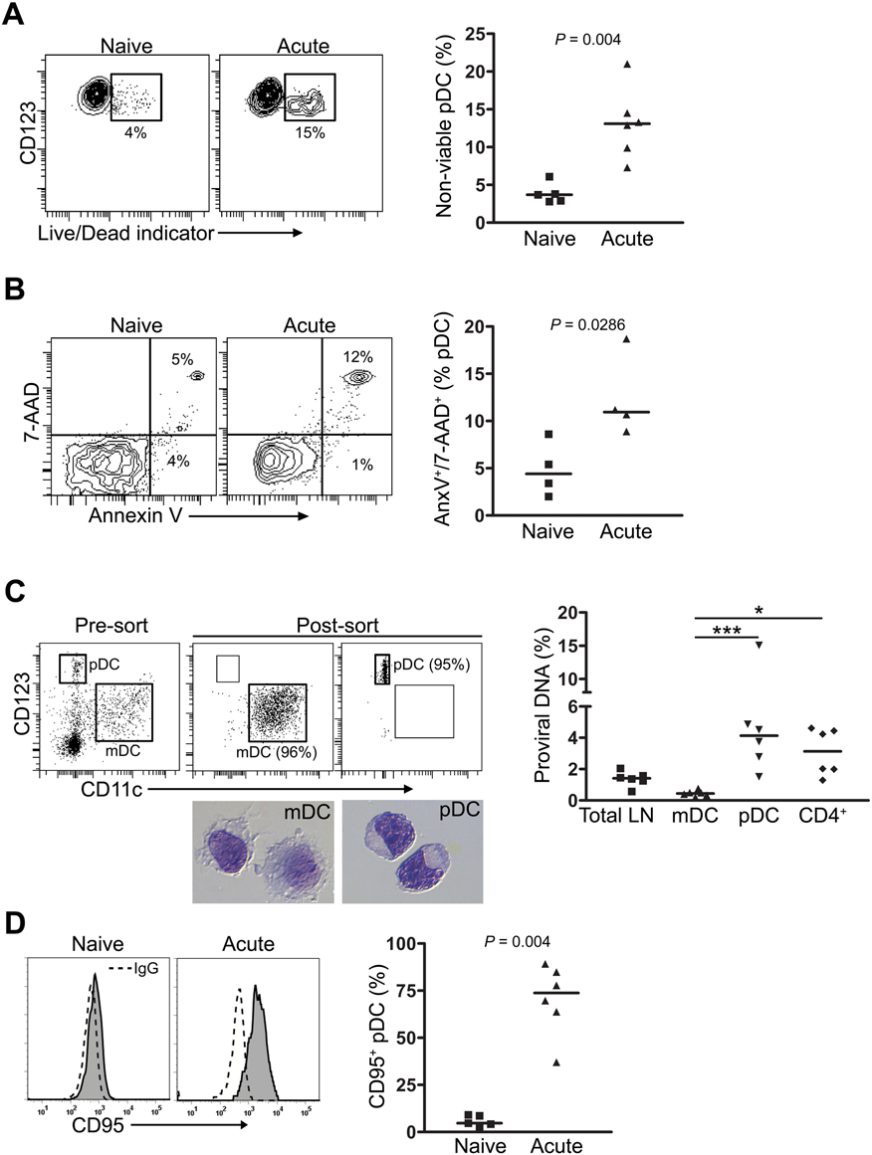
Activation, infection, and death of pDC in SIV–infected lymph nodes. (A) Left: Contour plots demonstrating staining of lymph node pDC from SIV-naïve and SIV-infected animals with Live/Dead viability dye. Numbers represent the percentage of non-viable pDC. Right: Percent of non-viable pDC in lymph nodes of SIV-naive animals and animals at 14 days post-infection. Symbols represent individual animals and horizontal lines represent medians. (B) Left: Representative contour plots demonstrating staining to define early apoptotic (Annexin V^+^/7-AAD^−^) and late apoptotic/necrotic (Annexin V^+^/7-AAD^+^) pDC from lymph nodes. Numbers represent the percent of cells in respective quadrants. Right: Percent of late apoptotic/necrotic pDC in lymph nodes from SIV-naive animals and animals at 14 days after infection. Symbols represent individual animals and horizontal lines represent medians. (C) Left: Representative dot plots demonstrating pDC and mDC in the Lineage^−^ HLA-DR^+^ gate prior to and after sorting from the axillary lymph node of a monkey 14 days after infection. Numbers in parentheses indicate the purity of mDC and pDC. Representative images of sorted mDC and pDC following modified Wright-Giemsa staining are also shown. Right: Frequency of total lymph node (LN) cells or sorted mDC, pDC and CD4^+^ T cells containing proviral DNA as determined by quantitative PCR. *P<0.05, ***P<0.001. Symbols represent individual animals and horizontal lines represent medians. (D) Left: Representative histograms demonstrating CD95 expression (solid histogram) on pDC compared to isotype control (dotted line) in naïve and SIV-infected lymph nodes. Right: Percent of lymph node pDC expressing CD95 in SIV-naive macaques and animals with acute SIV infection. Symbols represent individual animals and horizontal lines represent medians.

The overall loss of pDC from lymph nodes despite their massive recruitment strongly suggests that pDC are dying in these tissues during infection. To examine this directly, we first stained lymph node cell suspensions with antibodies to define pDC and then measured the proportion of cells that were dead based on staining with the amine-reactive viability dye. Nearly 15% of pDC from lymph nodes taken at 14 days post infection were non-viable as compared to 3% of pDC from naïve lymph nodes (Figure 5A). This increase was due to an increase in late apoptosis and/or necrosis based on pDC co-labeling with Annexin V and 7-AAD (Figure 5B). These data indicate that pDC resident in lymph nodes are more likely to be apoptotic or necrotic as a result of acute SIV infection. To determine whether direct virus infection could play a role in pDC death, we stained lymph node cell suspensions with antibodies and simultaneously sorted pDC as Live/Dead^−^ Lineage^−^ HLA-DR^+^ CD123^+^ CD11c^−^ cells and mDC as Live/Dead^−^ Lineage^−^ HLA-DR^++^ CD123^−^ CD11c^+^ [13] to at least 95% purity (Figure 5C). SIV *gag* DNA was then measured in total cellular DNA from these cells using *gag* primers and probe by quantitative PCR and the frequency of infected cells determined on the basis of *albumin* DNA quantity, as described in the Methods section [31]. In separate tubes we sorted CD4^+^ T cells as Live/Dead^−^ CD3^+^ CD4^+^ CD8^−^ cells and determined their frequency of SIV infection. These data revealed that more than 4% of lymph node pDC harbored SIV *gag* DNA at day 14, reaching up to 15% in one animal, a frequency that was slightly greater than SIV-infected CD4^+^ T cells and well above that of the unseparated lymph node fraction (Figure 5C). In contrast, mDC had almost undetectable levels of *gag* DNA (Figure 5C). The negligible infection of mDC confirms that the pDC result was not due to contaminating CD4^+^ T cells, as mDC and pDC were sorted simultaneously from the same tubes. To investigate whether indirect mechanisms of pDC death may also contribute to the significant cell death seen in lymph nodes, we measured expression of known pro-apoptotic tumor necrosis factor (TNF) family members CD95 (Fas), TNF-related apoptosis-inducing ligand (TRAIL) and the TRAIL receptor death receptor (DR) 5 by flow cytometry. These data revealed that lymph node pDC from SIV-naïve monkeys had almost undetectable expression of CD95, whereas CD95 was uniformly expressed by pDC from SIV-infected lymph nodes, indicative of activation [36] (Figure 5D). However, we were not able to detect differences in apoptosis of lymph node pDC from uninfected or infected monkeys after incubation with CD178 (Fas ligand; data not shown), consistent with earlier reports [17]. No expression of TRAIL or DR5 was detected on lymph node pDC regardless of SIV infection status (data not shown). These data show that pDC in lymph nodes of monkeys with acute SIV infection are activated and have increased death through apoptosis and necrosis, and that a significant fraction of pDC are infected with virus.

### Blood and lymph node pDC have largely normal function in acute SIV infection

**Figure 6:**
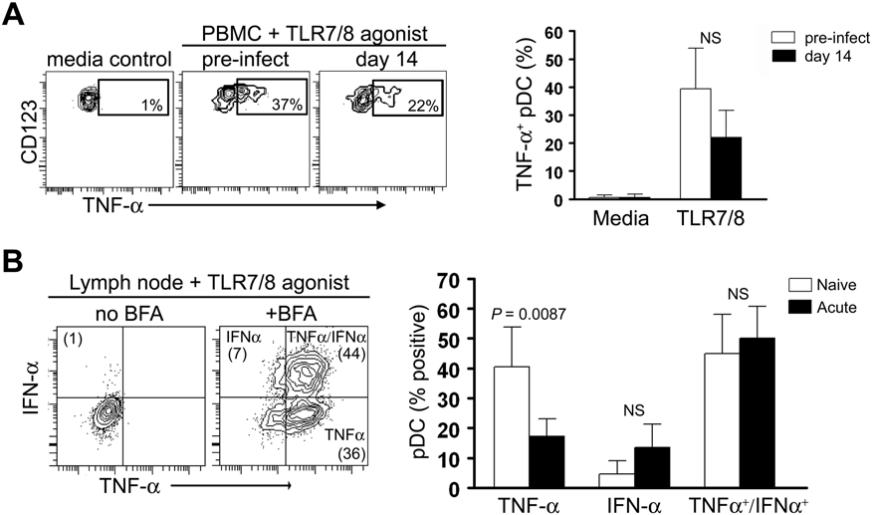
pDC retain largely normal function in response to TLR7 stimulation during acute SIV infection. (A) Left: Contour plots demonstrating the gating strategy used to identify TNF-α^+^ pDC in PBMC from SIV-naïve and SIV-infected monkeys following stimulation with 3M-007. Numbers represent the percentage of TNF-α^+^ pDC within the indicated gates. Right: Percent of TNF-α^+^ pDC in PBMC from SIV-naïve and SIV-infected monkeys in response to media or 3M-007. Bars represent the median and error bars the 95% confidence interval. (B) Left: Representative contour plots demonstrating the gating strategy used to identify TNF-α^+^, IFN-α^+^, or TNF-α^+^/ IFN-α^+^ pDC in lymph nodes following 3M-007 stimulation in the absence or presence of Brefeldin A (BFA). Numbers in parentheses represent the percentage of cells expressing the respective cytokines. Right: Percent of lymph node pDC from SIV-naïve and SIV-infected monkeys expressing TNF-α, IFN-α, or both in response to TLR7/8 agonist. Bars represent the median and error bars the 95% confidence interval. NS = not significant.

We next examined the function of pDC in blood and lymph nodes during acute SIV infection. Given that SIV and HIV stimulate pDC in large part through TLR7 [22],[34], we analyzed pDC function by stimulating cells with the TLR7/8 agonist 3M-007, as in earlier experiments. We did a short-term stimulation of unseparated PBMC taken before infection or at day 14 post infection with 3M-007 and determined expression of TNF-α by flow cytometry, gating on CD123^+^ pDC (Figure 6A). Between 20% and 40% of pDC in blood produced TNF-α in response to TLR7 stimulation regardless of infection status (Figure 6A). We next stimulated CD20-depleted lymph node cells taken from SIV-naïve monkeys or monkeys at 14 days post infection in the same manner, this time analyzing cells for expression of IFN-α and TNF-α. Approximately half of all lymph node pDC produced both TNF-α and IFN-α in response to this stimulation regardless of whether lymph nodes were taken from SIV-naïve or SIV-infected monkeys, whereas endogenous cytokine production in the absence of stimulation was negligible (Figure 6B and data not shown). Interestingly, the small proportion of pDC producing IFN-α alone was undiminished by SIV infection, although lymph node pDC from SIV-infected monkeys had a reduced capacity to produce TNF-α alone (Figure 6B). These findings indicate that despite a net loss of pDC the remaining cells in blood and lymph node retained largely normal functional responses to TLR7 stimulation during primary SIV infection.

## Discussion

In this study we used rhesus macaques infected with the pathogenic SIVmac251 strain to model kinetics of the acute pDC response to HIV infection of humans. We combined enumeration of cells in blood and lymphoid tissues with BrdU incorporation and Ki-67 staining to document pDC production and trafficking. We first confirmed that blood pDC do not cycle upon activation and that lymph node pDC isolated during acute SIV infection are not actively dividing, consistent with findings in the mouse [5]. Hence we were able to use markers of cell division to identify pDC in blood and lymph node as recent emigrants from bone marrow.

Our findings reveal a rapid and highly dynamic pDC response to SIV infection with mobilization of cells into blood occurring as soon as 3 days after intravenous virus challenge based on absolute pDC determinations. By 11 days after infection around 20% of blood pDC had mobilized from bone marrow in the previous 24-hour period as determined by BrdU incorporation, and this reached 60% by day 14 after four administrations of BrdU. In fact this is likely to be an underestimate of the extent of pDC mobilization given that BrdU has a half-life of only 2 hours in the circulation [37]. This mobilization is likely the result of increased systemic TNF-α [38] which has been shown to control pDC mobilization in murine models [4]. By days 10 to 14 of infection the number of pDC in blood had dramatically declined coincident with massive recruitment of pDC to lymph nodes, with the proportion of lymph node pDC that had recently been released from bone marrow being 10 to 20-fold greater than that of naïve monkeys by 14 days post infection. Rapid recruitment is consistent with TNF-α–driven expression of CXCL9 and upregulation of CCL19 in lymph nodes [4],[23],[24],[39] and with induction of CCR7 on activated pDC in the circulation [21],[22], leading to transmigration across high endothelial venules and localization to the T-cell rich paracortex. These data show that the inflammatory response in acute SIV infection is highly effective at inducing the coordinated mobilization and recruitment of large numbers of pDC into blood and lymph nodes.

Paradoxically, pDC recruitment was occurring as pDC numbers in lymph nodes were dramatically declining, indicating that cells were dying at a rate faster than they could be replenished. Rapid influx of pDC to lymph nodes at a time when virus is highly concentrated in these tissues [40] would increase pDC interaction with virus-infected CD4^+^ T cells in the paracortex, facilitating cell-to-cell fusion and death by apoptosis and necrosis [17]. Consistent with this possibility, we found around 15% of pDC undergoing late apoptosis and/or necrosis in acutely infected lymph nodes. In addition, high virus levels in lymph nodes would promote direct infection of pDC, and we show using highly purified cell populations that a significant fraction of pDC harbored proviral DNA, indicating that virus infection had occurred and proceeded at least to the point of reverse transcription. While the frequency of infected pDC was relatively modest at 4% it was similar to that of CD4^+^ T cells, the principle cells infected in lymph nodes at this time [41], suggesting that pDC are a significant target of virus infection. Previous reports have described Gag-expressing pDC in lymphoid tissues and pDC harboring proviral DNA in blood of HIV-infected individuals [25],[26], but up to now the efficiency of pDC infection *in vivo* was not known. By contrast, the frequency of infected mDC was almost below detection, indicating that these cells may not be a significant source of virus in lymph nodes [42]. While limited cell numbers did not allow us to directly determine whether infection resulted in cell death, *in vitro* data indicate that pDC infection concurrent with activation through CD40L results in giant cell formation and death [29]. *In vivo*, activated CD4^+^ T cells that engage infected pDC in the lymph node paracortex would provide CD40L, and Gag-expressing CD123^+^ multinucleated giant cells have been identified in HIV-infected lymphoid tissues [26], consistent with the notion of virus-induced pDC death.

Our data support the hypothesis that the physiologic response of pDC to enter inflamed lymph nodes is actually deleterious in pathogenic immunodeficiency virus infection as it brings pDC into an environment rich with virus, virus-infected cells and pro-apoptotic factors that lead to cell death. The continued loss of pDC would disrupt normal homeostasis leading to enhanced mobilization of cells into blood and influx into lymph nodes in an attempt to replenish the pDC pool, exacerbating the effect. Depletion of pDC from lymphoid tissues subsequent to recruitment is likely to have a significant impact on virus control at a number of levels. Activated pDC directly suppress HIV replication in CD4^+^ T cells [17] and can stimulate antigen-specific CD4^+^ T cell responses [21]. Virus-exposed pDC induce maturation of mDC *in vitro*, and pDC cooperate with mDC in the induction of virus-specific cytotoxic T cell responses *in vivo* [3],[21]. In addition, type I IFN production by pDC inhibits HIV replication [19].

pDC depletion from lymph nodes is found in chronically HIV-infected individuals [43] and in advanced pathogenic SIV infection of rhesus and pig-tailed macaques [13],[14]. Our data showing similar depletion in acute SIVmac251 infection of Indian rhesus macaques would appear to conflict with studies from other pathogenic SIV models, including SIVsm infection in the same species and SIVmac251 infection of Mauritius cynomolgus macaques, which indicate that acute infection is associated with increased numbers of pDC in lymph nodes [16],[22]. In SIV-infected cynomolgus monkeys in particular increases in pDC number in lymph nodes were evident at 38 days and even 9 months post infection [16]. It is interesting to note that pathogenicity of SIVmac251 is attenuated in Mauritius cynomolgus macaques relative to Indian rhesus macaques, associated with a more pronounced virus-specific cellular immune response at 2 weeks post infection [44]. It is possible therefore that enhanced immune control of virus replication occurring in lymphoid tissues of infected cynomolgus macaques results in reduced exposure of newly recruited pDC to virus and virus-infected cells leading to a reduction in pDC death. Hence the divergent response of pDC in lymph nodes as a result of infection could potentially be an early indicator of differential pathogenicity of SIVmac251 in these two species. Our data raise concerns over the hypothesis that muted pDC recruitment to lymph nodes is integral to the lack of disease in nonpathogenic SIV infection of sooty mangabeys [22], especially given that similar studies in nonpathogenic SIV infection of African green monkeys reveal significant pDC influx and activation in lymph nodes during the acute phase of infection [45].

In our study pDC from SIV-infected lymph nodes uniformly expressed CD95, and in mice exposed to inflammatory and microbial stimuli CD95 expression induced on activated pDC is linked to apoptotic cell death [36]. However, while CD95/CD178 interactions play a role in CD4^+^ T cell loss in HIV and SIV infection [46],[47] the role for this pathway in pDC death is yet to be established [17]. We were not able to detect TRAIL expression on pDC isolated from lymph nodes of SIV-infected monkeys, despite recent studies indicating that HIV induces expression of TRAIL on pDC in an IFN-α dependent manner [48]. Serum levels of TRAIL are increased in SIV-infected macaques as in HIV-infected humans [49],[50], and high expression of TRAIL mRNA in lymph nodes of SIV-infected macaques is reduced by antiretroviral therapy [51]. However, TRAIL expression is not limited to pDC, as CD4^+^ T cells from SIV-infected rhesus macaques express significant levels of TRAIL as do rhesus monocytes and monocyte-derived DC exposed to SIV *in vitro* [50]. More studies are needed to clarify which cell types express TRAIL in lymphoid tissues during HIV and SIV infection and the functional consequences of this expression.

pDC production in bone marrow was not significantly affected by acute SIV infection in this study, although additional studies in a larger cohort of animals are needed to confirm these findings due to the marked variability in pDC number in aspirates. A similar wide variation in pDC number was noted in bone marrow aspirates analyzed from SIV-naïve pig-tailed macaques [32]. No prior reports to our knowledge have investigated the effects of SIV infection on pDC production from bone marrow. Studies in SIV-infected macaques indicate that virus does not infect CD34^+^ hematopoietic stem cells *in vivo* [52], although defects in bone marrow hematopoiesis that significantly impact upon the generation of CD4^+^ T cells from CD34^+^ progenitors *ex vivo* have been demonstrated [53]. Our study evaluated pDC kinetics in bone marrow only up to 12 days post infection and it is possible that hematopoietic suppression may not be occurring at this very early stage. Indeed, bone marrow suppression as measured by a decrease in colony forming units is only evident beginning at 21 days post infection of macaques with a pathogenic simian/human immunodeficiency virus [54].

Reduction in circulating type I IFN has been observed in HIV-infected individuals and is generally thought to be due to impaired pDC function [8]–[10],[12],[55],[56]. PBMC that were stimulated *in vitro* with herpes simplex virus had significantly lower type I IFN in culture supernatants when isolated from patients with primary HIV infection relative to controls, and this was associated with lower numbers of blood pDC [12]. In contrast, in our study pDC from blood and lymph nodes in monkeys 14 days after SIVmac251 infection were largely normal in their response to TLR7 stimulation, with lymph node pDC producing TNF-α and IFN-α together at high levels that were indistinguishable from controls. This discrepancy may be a consequence of experiments being performed at different stages of infection, as in the study of primary HIV infection individuals had a median of 51 days after the presumed date of infection [12]. Studies using intracellular cytokine production to directly examine pDC function revealed that markedly reduced pDC production of IFN-α was evident in HIV-infected individuals with high virus burden and low CD4^+^ T cell counts, suggesting that pDC dysfunction worsens with advanced disease [9]. Whether IFN-α production by the limited number of surviving pDC in acute infection is sufficient to induce chronic CD4^+^ T cell activation in lymph nodes, as has recently been proposed [22],[57], remains to be determined.

While we were not able to sample mucosal tissues in the current study, it is possible that these tissues provide a significant sink for pDC that have been mobilized into blood in acute SIV infection, especially given that viral replication and CD4^+^ T cell depletion occur to a large extent in gut mucosa [31],[58],[59]. In addition, vaginal SIV infection rapidly induces a local inflammatory response with pronounced type I IFN production [40],[60], and similar responses occurring subsequent to mucosal viral infection in the mouse are associated with pDC recruitment and activation that suppresses virus replication [61]. The relationship between pDC and mucosa in the context of SIV infection is likely to be important in disease pathogenesis and needs to be addressed.

## Materials and Methods

### Animals, virus infection, and sample collection

Six adult female Indian-origin rhesus macaques (*Macaca mulatta*) were infected by intravenous inoculation with 1,000 TCID_50_ SIVmac251 that had been grown in CEMx174 cells [62] (kindly provided by Preston Marx, Tulane National Primate Research Center) and compared to a cohort of six SIV-naive controls. Blood samples were collected in either EDTA or ACD four to six times prior to infection including day 0 and on days 3, 6, 10, 11, 12, 13 and 14 post-infection. BrdU (Sigma) was prepared in Ca^2+^/Mg^2+^-free HBSS at 10 mg/ml, filter-sterilized and administered by intravenous injection at 30 mg/kg at 24-hour intervals for 4 doses starting on day 10 post-infection. Parallel BrdU administration was given to two SIV-naïve monkeys. Bone marrow aspirates were obtained at least 30 days prior to and on day 12 post-SIV infection from the medullary cavity of alternating femurs using a Jamshidi bone marrow biopsy needle flushed with heparin. SIV-infected animals were sacrificed on day 14 post-infection and the two SIV-naive animals receiving BrdU were sacrificed 24 hour after the final drug dose. All animals underwent transcardiac saline perfusion at the time of sacrifice to ensure removal of peripheral blood from tissues. All experiments were performed using protocols approved by appropriate institutional regulatory committees.

### Sample processing

PBMC were isolated by density gradient centrifugation over Ficoll. Alternatively, blood samples were lysed of red blood cells using ACK lysing buffer for isolation of PBL. Lymph node single-cell suspensions were generated and depleted of CD20^+^ B cells as previously described [13]. Bone marrow aspirates were passed over a 70 µm nylon cell strainer to remove spicules and diluted 1∶1 with PBS prior to erythrocyte lysis and counting using trypan blue exclusion. All blood, lymph node and bone marrow preparations were frozen in medium containing 90% FBS and 10% DMSO (Sigma) using a controlled-rate freezer (CryoMed) and stored in liquid nitrogen. Viral RNA levels in plasma were determined by real-time PCR using an ABI Prism 7900H sequence detection system (Applied Biosystems) using reverse-transcribed viral RNA as templates, as described [63].

### Flow cytometry, cell enumeration, and cytokine expression

All antibodies were purchased from BD Biosciences unless noted otherwise. For the majority of analyses cells were stained with antibodies to Lineage markers [CD3 (clone SP34-2), CD14 (M5E2), and CD20 (2H7, eBioscience)], CD45 (D058-1283), HLA-DR (G46-6), and CD123 (7G3) with or without an amine-reactive Live/Dead cell viability dye (Invitrogen) using mutually-exclusive fluorochrome conjugates. In some experiments cells were co-stained with Ab to CD95 (DX2) or with 5.0 µg/mL 7-AAD and Annexin V (BD Biosciences). Alternatively, labeled cells were fixed and permeabilized with Cytofix/Cytoperm (BD Biosciences) and incubated with Ab to Ki-67 (B56). To detect *in vivo* BrdU incorporation labeled cells were fixed with 4% formaldehyde prior to incubation with DNase I (Roche) in 0.1% saponin and incubation with antibody to BrdU (B44). In some experiments PBMC and lymph node cells were pulsed with 10 µM BrdU *ex vivo* for 4 hours prior to staining with antibody to BrdU as per manufacturer's instructions (BrdU Flow Kit, BD Pharmingen). Absolute numbers of cells were determined using a modified single-platform quantification assay as described [30]. Briefly, the number of CD45^+^ mononuclear cells and CD4^+^ T cells in blood were determined by staining 50 µL of whole blood with antibodies to CD3, CD4 (L200) and CD45 using TruCOUNT tubes (BD Biosciences) as per the manufacturer's recommendations. For pDC quantification PBL were stained with antibodies to define pDC, and the absolute number of pDC/µL blood was then calculated by multiplying the percentage of pDC within the CD45^+^ fraction by the absolute number of CD45^+^ cells calculated using whole blood in TruCOUNT tubes. A similar method was used to calculate mononuclear cells and pDC in bone marrow aspirates. For analysis of intracellular cytokine expression, PBMC were stimulated for 5 hours in media alone or with 10 µM of the TLR7/8 agonist 3M-007 (3M Pharmaceuticals) in the presence of 10 µg/mL brefeldin A (Sigma) for the final 3 hours. Alternatively, CD20-depleted lymph node cells were stimulated for 12 hours for convenience with 10 µM 3M-007 agonist in the presence or absence of 10 µg/mL brefeldin A included after 1 hour. Cells were stained with surface-labeling antibodies and fixed and permeabilized as above prior to incubation with antibodies to TNF-α (MAb11) and/or IFN- α (MMHA-2, PBL Biomedical Laboratories). Ab to IFN- α was conjugated to Alexa 647 using Zenon labeling technology according to the manufacturer's protocol (Invitrogen). In some experiments PBMC were stimulated with 10 µM 3M-007 in the presence of 20 ng/ml recombinant human IL-3 (R&D Systems) for 20 hours prior to pulsing with BrdU as above. Flow cytometry data was acquired using a BD LSR II or FACS Aria flow cytometer and analyzed with BD FACS Diva software (version 5.0.2) and histogram overlays generated using FlowJo version 7.2.4 (Tree Star, Inc.).

### Cell sorting

Previously cryopreserved lymph node cell suspensions were thawed in the presence of DNAse, strained and then stained with antibodies to Lineage markers, HLA-DR, CD123 and CD11c (S-HCL-3) along with the Live/Dead viability dye. Between 5,000 and 10,000 viable Lin^−^ HLA-DR^+^ CD123^+^ CD11c^−^ pDC and Lin^−^ HLA-DR^++^ CD123^−^ CD11c^+^ mDC were sorted simultaneously using a BD FacsAria flow cytometer, making sure to exclude doublets on the basis of forward scatter and side scatter height and width parameters. In these sorting experiments we used a broad Lineage^−^ HLA-DR^+/++^ gate to encompass both DC subsets as described previously [13]. For purification of CD4^+^ T cells, lymph node suspensions were stained with antibodies to CD3, CD4 and CD8 (B9.11, Immunotech) and 50,000 viable CD3^+^CD4^+^CD8^−^ cells sorted as above. Purified cells were deposited into tubes containing lysis buffer for DNA isolation or into tubes containing media followed by centrifugation onto slides (Cytospin 3, Shandon). Slides were fixed and stained using a modified Wright-Giemsa stain (Hema 3, Fisher) and imaged using an Olympus BX51 light microscope.

### Quantification of SIV proviral DNA

Cellular DNA from sorted cells was isolated using DNAeasy blood and tissue kit (Qiagen). For quantitative PCR analysis, a plasmid was first generated by inserting sequences of SIVsmB7 long terminal repeat and SIVmac239 *gag* in the pUC19 plasmid along with macaque albumin cDNA. This plasmid was validated against serial dilutions of B7 cells containing a single integrated copy of SIVsmB7 [64] by using TaqMan real-time PCR with primers 5′-TTGAGCCCTGGGAGGTTCT-3′ and 5′-CCAAGTGCTGGTGAGAGTCTAG-3′, and probe 5′-CAGCACTAGCAGGTAGAGCCTGGGTGTTC-3′, and used as a standard for calculating the frequency of infection in sorted cells. SIVmac251 *gag* copy number in each DNA sample was quantified by comparison with serial dilutions of the plasmid standard using TaqMan real-time PCR using primers 5′-GCCAGGATTTCAGGCACTGT-3′ and 5′-GCTTGATGGTCTCCCACACAA-3′, and the probe 5′-AAGGTTGCACCCCCTATGACATTAATCAGATGTTA-3′ specific for sequences of *gag* that are conserved across SIV strains. In parallel, the albumin gene copy number was determined to quantify the number of cells loaded in each reaction using primers 5′-TGCATGAGAAAACGCCAGTA-3′ and 5′-AGCATGGTCGCCTGTTCAC-3′ and probe 5′-AAAGTCACCAAATGCTGCACGGAATCC-3′. Reactions were performed in duplicate using TaqMan Universal mix kit (Applied Biosystems), 200 nM of each primer and 125 nM probe, at 95°C for 10 minutes, with 40 cycles at 95°C for 15 seconds and at 60°C for 1 minute using the Prism 7900H sequence detection system (Applied Biosystems).

### Statistical analysis

Differences between pre and post-infection time points were determined using the nonparametric Wilcoxon signed-rank test. Cross-sectional analysis of SIV-naïve and animals with acute SIV infection were performed using the nonparametric Mann-Whitney *U* test. Correlations were determined using the non-parametric Spearman rank test. Multiple comparisons between the frequencies of SIV infection in sorted populations were performed using the Kruskal-Wallis test followed by Dunn's post test. All statistical comparisons were considered significant when two-tailed *P*<0.05.

## Supporting Information

Figure S1 Intravenous inoculation with SIVmac251 produces high plasma virus load and blood CD4^+^ T cell loss.
(0.12 MB PDF)

Figure S2 Rhesus macaque pDC from blood and lymph node fail to proliferate following *ex vivo* activation or *in vivo* SIV infection.
(0.10 MB PDF)
