# Supplementary material for: Rapid Influx and Death of Plasmacytoid Dendritic Cells in Lymph Nodes Mediate Depletion in Acute Simian Immunodeficiency Virus Infection
Source: PLoS Pathog. 2009 May 8;5(5):e1000413. doi: 10.1371/journal.ppat.1000413 (PMC2671605; doi:10.1371/journal.ppat.1000413)
Supplement: Figure S1 — Intravenous inoculation with SIVmac251 produces high plasma virus load and blood CD4+ T cell loss. (0.12 MB PDF) [file ppat.1000413.s001.pdf]

Figure S1

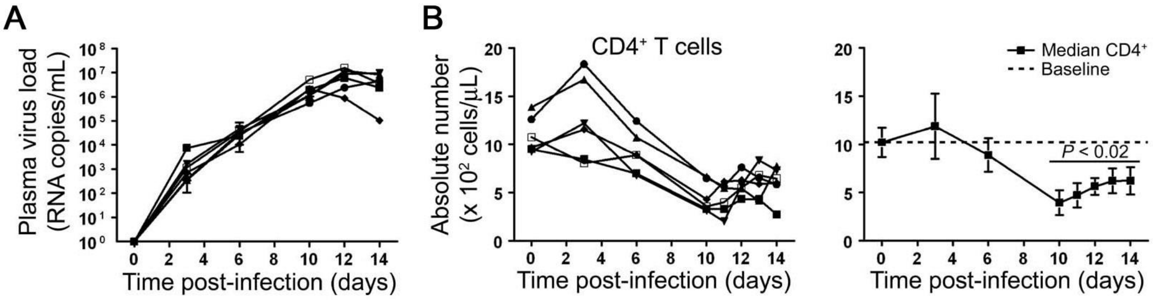

Intravenous inoculation with SIVmac251 produces high plasma virus load and blood $CD4^+$ T cell loss. (**A**) Plasma virus load was determined at the indicated times following inoculation with 1,000 $TCID_{50}$ SIVmac251 using real-time RT-PCR as described in Methods. Symbols represent individual animals and error bars the 95% confidence interval of samples run in duplicate. (**B**) Changes in the absolute number of $CD4^+$ T cells in blood for all animals during acute SIV infection as determined using the TruCOUNT assay. Day 0 represents the median number of cells from at least 4 independent measurements prior to infection including day 0. Symbols represent individual animals (left) and the group median $\pm$ 95% confidence interval (right). The dashed line represents the overall group median baseline cell number/µL of blood. Each time point starting on day 10 was significantly decreased compared to pre-infection.
