# Supplementary material for: Rapid Influx and Death of Plasmacytoid Dendritic Cells in Lymph Nodes Mediate Depletion in Acute Simian Immunodeficiency Virus Infection
Source: PLoS Pathog. 2009 May 8;5(5):e1000413. doi: 10.1371/journal.ppat.1000413 (PMC2671605; doi:10.1371/journal.ppat.1000413)
Supplement: Figure S2 — Rhesus macaque pDC from blood and lymph node fail to proliferate following ex vivo activation or in vivo SIV infection. (0.10 MB PDF) [file ppat.1000413.s002.pdf]

Figure S2

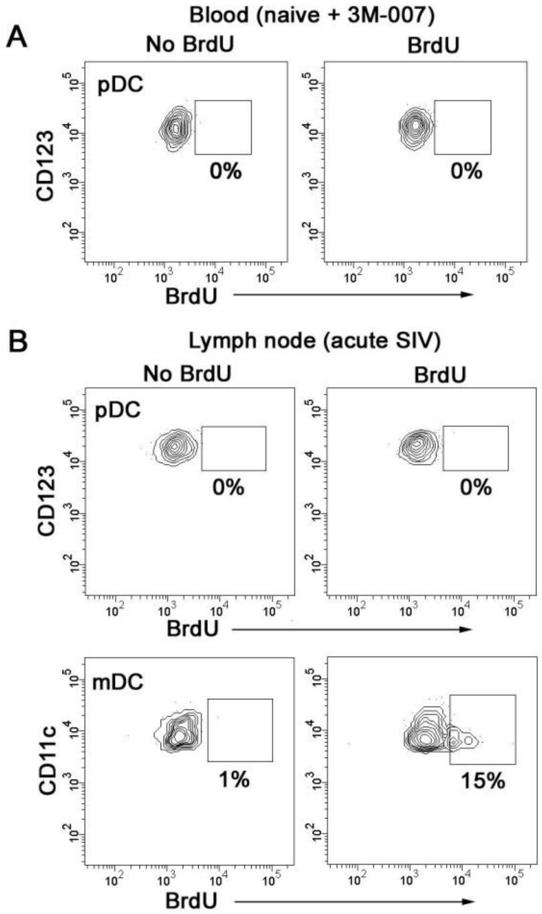

Rhesus macaque pDC from blood and lymph node fail to proliferate following *ex vivo* activation or *in vivo* SIV infection. (**A**) PBMC from a SIV-naïve monkey were cultured for 20 hours in the presence of 20 ng/ml IL-3 and 10 μM 3M-007 with and without a pulse of 10 μM BrdU in the last 4 hours. Cells were stained with viability dye and antibodies as described in Materials and Methods and analyzed by flow cytometry for evidence of BrdU incorporation. Shown are live $CD123^+$ pDC within the $Lineage^-$ $HLA\text{-}DR^+$ gate. (**B**) Lymph node cells harvested from a rhesus macaque 14 days after intravenous inoculation with SIVmac251 were cultured in the presence of 20 ng/ml IL-3 for 6 hours with and without a pulse of 10 μM BrdU in the last 4 hours. Cells were harvested and stained as above. Shown are live $CD123^+$ pDC (top) and $CD11c^+$ mDC (bottom) within the $Lineage^-$ $HLA\text{-}DR^{+/++}$ gate. Numbers represent the percentage of $BrdU^+$ cells within each gate.
